# Subclass-based multi-task learning for Alzheimer's disease diagnosis

**DOI:** 10.3389/fnagi.2014.00168

**Published:** 2014-08-07

**Authors:** Heung-II Suk, Seong-Whan Lee, Dinggang Shen, The Alzheimers Disease Neuroimaging Initiative

**Affiliations:** ^1^Department of Radiology, Biomedical Research Imaging Center, University of North Carolina at Chapel HillChapel Hill, NC, USA; ^2^Department of Brain and Cognitive Engineering, Korea UniversitySeoul, Republic of Korea

**Keywords:** Alzheimer's disease, mild cognitive impairment, neuroimaging analysis, feature selection, *K*-means clustering

## Abstract

In this work, we propose a novel subclass-based multi-task learning method for feature selection in computer-aided Alzheimer's Disease (AD) or Mild Cognitive Impairment (MCI) diagnosis. Unlike the previous methods that often assumed a unimodal data distribution, we take into account the underlying multipeak^1^ distribution of classes. The rationale for our approach is that it is highly likely for neuroimaging data to have multiple peaks or modes in distribution, e.g., mixture of Gaussians, due to the inter-subject variability. In this regard, we use a clustering method to discover the multipeak distributional characteristics and define subclasses based on the clustering results, in which each cluster covers a peak in the underlying multipeak distribution. Specifically, after performing clustering for each class, we encode the respective subclasses, i.e., clusters, with their unique codes. In encoding, we impose the subclasses of the same original class close to each other and those of different original classes distinct from each other. By setting the codes as new label vectors of our training samples, we formulate a multi-task learning problem in a ℓ_2,1_-penalized regression framework, through which we finally select features for classification. In our experimental results on the ADNI dataset, we validated the effectiveness of the proposed method by improving the classification accuracies by 1% (AD vs. Normal Control: NC), 3.25% (MCI vs. NC), 5.34% (AD vs. MCI), and 7.4% (MCI Converter: MCI-C vs. MCI Non-Converter: MCI-NC) compared to the competing single-task learning method. It is remarkable for the performance improvement in MCI-C vs. MCI-NC classification, which is the most important for early diagnosis and treatment. It is also noteworthy that with the strategy of modality-adaptive weights by means of a multi-kernel support vector machine, we maximally achieved the classification accuracies of 96.18% (AD vs. NC), 81.45% (MCI vs. NC), 73.21% (AD vs. MCI), and 74.04% (MCI-C vs. MCI-NC), respectively.

## 1. Introduction

As the population is aging, the brain disorders under the broad category of dementia such as Alzheimer's Disease (AD), Parkinson's disease, etc. have been becoming great concerns around the world. In particular, AD, characterized by progressive impairment of cognitive and memory functions, is the most prevalent cause of dementia in elderly people. According to a recent report by Alzheimer's Association, the number of AD patients is significantly increasing every year, and 10–20 percent of people aged 65 or older have Mild Cognitive Impairment (MCI), a prodromal stage of AD (Alzheimer's Association, 2012). While there is no cure for AD to halt or reverse its progression, it has been of great importance for early diagnosis and prognosis of AD/MCI in the clinic, due to the symptomatic treatments available for a limited period in the spectrum of AD.

To this end, there have been a lot of studies to discover biomarkers and to develop a computer-aided diagnosis system with the help of neuroimaging such as Magnetic Resonance Imaging (MRI) (Cuingnet et al., 2011; Davatzikos et al., 2011; Wee et al., 2011; Zhou et al., 2011; Li et al., 2012; Zhang et al., 2012), Positron Emission Tomography (PET) (Nordberg et al., 2010), functional MRI (fMRI) (Greicius et al., 2004; Suk et al., 2013b). It has been also shown that fusing the complementary information from multiple modalities, e.g., MRI+PET, helps enhance the diagnostic accuracy (Fan et al., 2007; Perrin et al., 2009; Kohannim et al., 2010; Walhovd et al., 2010; Cui et al., 2011; Hinrichs et al., 2011; Zhang et al., 2011; Wee et al., 2012; Westman et al., 2012; Yuan et al., 2012; Zhang and Shen, 2012; Suk and Shen, 2013).

However, from a computational modeling perspective, while the feature dimension of those neuroimaging is high in nature, we have a very limited number of observations/samples available. This so-called “small-*n*-large-*p*” problem (Fort and Lambert-Lacroix, 2005) has been of a great challenge in the field to build a robust model that can correctly identify a clinical label of a subject, e.g., AD, MCI, Normal Control (NC). For this reason, reducing the feature dimensionality, by which we can mitigate the overfitting problem and improve a model's generalizability, has been considered as a prevalent step in building a computer-aided AD diagnosis system as well as neuroimaging analysis (Mwangi et al., 2013).

In general, we can broadly categorize the approaches in the literature that aimed at lowering the feature dimensionality into feature-dimension reduction and feature selection. The methods of feature-dimension reduction find a mapping function that transforms the original feature space into a new low-dimensional space. Principal Component Analysis (PCA) and Linear Discriminant Analysis (LDA) (Martinez and Kak, 2001) are the representative methods of this category and to date, thanks to their computational efficiency, they have been the most widely used in various fields. The PCA finds a mapping function through which it still includes a large portion of the information in samples. Meanwhile, the LDA finds a transformation function that maps the original high-dimensional samples into the dimension-reduced ones by jointly maximizing the variance between classes and minimizing the variance within classes using a Fisher's criterion. However, since the learned projective functions in PCA or LDA are linear combinations of all the original features, it is often difficult to interpret the transformed features (Qiao et al., 2010). Clinically, it is unfavorable for the interpretational difficulty in neuroimaging analysis or classification.

Meanwhile, the feature selection approach that includes filter, wrapper, and embedded methods selects target-related features in the original feature space based on some criteria (Guyon and Elisseeff, 2003). Among these, the embedded methods, e.g., a ℓ_1_-penalized linear regression model (Tibshirani, 1994) and its variants (Roth, 2004), have recently attracted researchers due to their theoretical strengths and effectiveness in neuroimage analysis (Varoquaux et al., 2010; Fazli et al., 2011; de Brecht and Yamagishi, 2012; Suk et al., 2013a). In the ℓ_1_-penalized regression model, with a sparsity constraint using ℓ_1_-norm, many elements in the weighting coefficient vector become zero, thus the corresponding features can be removed. From a machine learning point of view, since the ℓ_1_-penalized linear regression model finds one weight coefficient vector that best regresses a target response vector, it is considered as a single-task learning. Hereafter, we use the terms of a ℓ_1_-penalized regression model and a single-task learning interchangeably.

The main limitation of the previous methods of PCA, LDA, and ℓ_1_-penalized regression model is that they consider a single mapping or a single weight coefficient vector in reducing the dimensionality. Here, if the underlying data distribution is not unimodal, e.g., mixture of Gaussians, then these methods would fail to find the proper mapping or weighting functions, and thus result in performance degradation. In this regard, Zhu and Martinez proposed a Subclass Discriminant Analysis (SDA) method (Zhu and Martinez, 2006) that first clustered samples of each class and then reformulated the conventional LDA by regarding clusters as subclasses. Recently, Liao and Shen applied the SDA method to segment prostate MR images and showed the effectiveness of the subclasses-based approach (Liao et al., 2013).

With respect to neuroimaging data, it is highly likely for the underlying data distribution to have multiple peaks due to the inter-subject variability (Fotenos et al., 2005; Noppeney et al., 2006; DiFrancesco et al., 2008). Here, it should be noted that although SDA was successfully applied to computer vision (Zhu and Martinez, 2006; Kim, 2010; Gkalelis et al., 2013) or medical image segmentation (Liao et al., 2013), as a variant of LDA, it still has an interpretational limitation. In this paper, we propose a novel method of feature selection for AD/MCI diagnosis by integrating the embedded method with the subclass-based approach. Specifically, we first divide each class into multiple subclasses by means of clustering, with which we can approximate the inherent multipeak data distribution of a class. Note that we regard each cluster as a subclass following Zhu and Martinez's work (Zhu and Martinez, 2006). Based on the clustering results, we encode the respective subclasses with their unique codes, for which we impose the subclasses of the same original class close to each other and those of different original classes distinct from each other. By setting the codes as new labels of our training samples, we finally formulate a multi-task learning problem in a ℓ_2,1_-penalized regression framework that takes into account the multipeak data distributions, and thus help enhance the diagnostic performances.

## 2. Materials and image processing

### 2.1. Subjects

In this work, we use the ADNI dataset publicly available on the web^2^. Specifically, we consider only the baseline MRI, 18-Fluoro-DeoxyGlucose (FDG) PET, and CerebroSpinal Fluid (CSF) data acquired from 51 AD, 99 MCI, and 52 NC subjects^3^. For the MCI subjects, they were further clinically subdivided into 43 MCI Converters (MCI-C), who progressed to AD in 18 months, and 56 MCI Non-Converters (MCI-NC), who did not progress to AD in 18 months. The demographics of the subjects are summarized in Table 1.

**Table 1:** **Demographic and clinical information of the subjects**.

	**AD**	**MCI converter**	**MCI non-converter**	**NC**
	**(*N* = 51)**	**(*N* = 43)**	**(*N* = 56)**	**(*N* = 52)**
Female/male	18/33	15/28	17/39	18/34
Age (Mean ± *SD*)	75.2 ± 7.4 [59–88]	75.7 ± 6.9 [58–88]	75.0 ± 7.1 [55–89]	75.3 ± 5.2 [62–85]
Education (Mean ± *SD*)	14.7 ± 3.6 [4–20]	15.4 ± 2.7 [10–20]	14.9 ± 3.3 [8–20]	15.8 ± 3.2 [8–20]
MMSE (Mean ± *SD*)	23.8 ± 2.0 [20–26]	26.9 ± 2.7 [20–30]	27.0 ± 3.2 [18–30]	29 ± 1.2 [25–30]
CDR (Mean ± *SD*)	0.7 ± 0.3 [0.5–1]	0.5 ± 0 [0.5–0.5]	0.5 ± 0 [0.5–0.5]	0 ± 0 [0–0]

(MMSE, Mini Mental State Examination, CDR, Clinical Dementia Rating, *N*, number of subjects, SD, Standard Deviation, [min-max]).

With regard to the general eligibility criteria in ADNI, subjects were in the age of between 55 and 90 with a study partner, who could provide an independent evaluation of functioning. General inclusion/exclusion criteria^4^ are as follows: (1) healthy normal subjects: Mini Mental State Examination (MMSE) scores between 24 and 30 (inclusive), a Clinical Dementia Rating (CDR) of 0, non-depressed, non-MCI, and non-demented; (2) MCI subjects: MMSE scores between 24 and 30 (inclusive), a memory complaint, objective memory loss measured by education adjusted scores on Wechsler Memory Scale Logical Memory II, a CDR of 0.5, absence of significant levels of impairment in other cognitive domains, essentially preserved activities of daily living, and an absence of dementia; and (3) mild AD: MMSE scores between 20 and 26 (inclusive), CDR of 0.5 or 1.0, and meets the National Institute of Neurological and Communicative Disorders and Stroke and the Alzheimer's Disease and Related Disorders Association (NINCDS/ADRDA) criteria for probable AD.

### 2.2. MRI and PET scanning

The structural MR images were acquired from 1.5T scanners. We downloaded data in Neuroimaging Informatics Technology Initiative (NIfTI) format, which had been pre-processed for spatial distortion correction caused by gradient non-linearity and B1 field inhomogeneity. The FDG-PET images were acquired 30–60 min post-injection, averaged, spatially aligned, interpolated to a standard voxel size, normalized in intensity, and smoothed to a common resolution of 8 *mm* full width at half maximum. CSF data were collected in the morning after an overnight fast using a 20- or 24-gauge spinal needle, frozen within 1 h of collection, and transported on dry ice to the ADNI Biomarker Core laboratory at the University of Pennsylvania Medical Center.

### 2.3. Image processing and feature extraction

The MR images were preprocessed by applying the typical procedures of Anterior Commissure (AC)-Posterior Commissure (PC) correction, skull-stripping, and cerebellum removal. Specifically, we used MIPAV software^5^ for AC-PC correction, resampled images to 256 × 256 × 256, and applied N3 algorithm (Sled et al., 1998) to correct intensity inhomogeneity. An accurate and robust skull stripping (Wang et al., 2013) was performed, followed by cerebellum removal. We further manually reviewed the skull-stripped images to ensure clean removal. Then, FAST in FSL package^6^ (Zhang et al., 2001) was used for structural MR image segmentation into three tissue types of Gray Matter (GM), White Matter (WM) and CSF. We finally pacellated them into 93 Regions Of Interests (ROIs) by warping Kabani et al.'s atlas (Kabani et al., 1998) to each subject's space via HAMMER (Shen and Davatzikos, 2002), although other advanced registration methods can also be applied for this process (Friston et al., 1995; Xue et al., 2006; Yang et al., 2008; Tang et al., 2009; Jia et al., 2010). In this work, we considered only GM for classification, because of its relatively high relatedness to AD/MCI compared to WM and CSF (Liu et al., 2012). Regarding FDG-PET images, they were rigidly aligned to the respective MR images, and then applied parcellation propagated from the atlas by registration.

For each ROI, we used the GM tissue volume from MRI, and the mean intensity from FDG-PET as features^7^, which are most widely used in the field for AD/MCI diagnosis (Davatzikos et al., 2011; Hinrichs et al., 2011; Zhang and Shen, 2012; Suk et al., 2013a). Therefore, we have 93 features from a MR image and the same dimensional features from a FDG-PET image. Here, we should note that although it is known that the regions of medial temporal and superior parietal lobes are mainly affected by the disease, we assume that other brain regions, although their relatedness to AD is not clearly investigated yet, may also contribute to the diagnosis of AD/MCI and thus we consider 93 ROIs in our study. In addition, we have three CSF biomarkers of Aβ_42_, *t*-tau, and *p*-tau as features.

## 3. Methods

In this section, we first briefly introduce the mathematical background of single-task and multi-task learning, and then describe a novel subclass-based multi-task learning method for feature selection in AD/MCI diagnosis.

### 3.1. Notations

Throughout the paper, we denote matrices as boldface uppercase letters, vectors as boldface lowercase letters, and scalars as normal italic letters, respectively. For a matrix **X** = [*x*_*ij*_], its *i*-th row and *j*-th column are denoted as **x**^*i*^ and **x**_*j*_, respectively. We further denote the Frobenius norm and ℓ_2,1_-norm of a matrix **X** as $\|X{\|}_{F}=\sqrt{\sum_{i}\|x^{i}{\|}_{2}^{2}}=\sqrt{\sum_{j}\|x_{j}{\|}_{2}^{2}}$ and $\|X{\|}_{2,1}=\sum_{i}\|x^{i}{\|}_{2}=\sum_{i}\sqrt{\sum_{j}x_{ij}^{2}}$, respectively, and the ℓ_1_-norm of a vector as ‖**w**‖_1_ = ∑_*i*_|*w*_*i*_|.

### 3.2. Background

**Figure 1:**
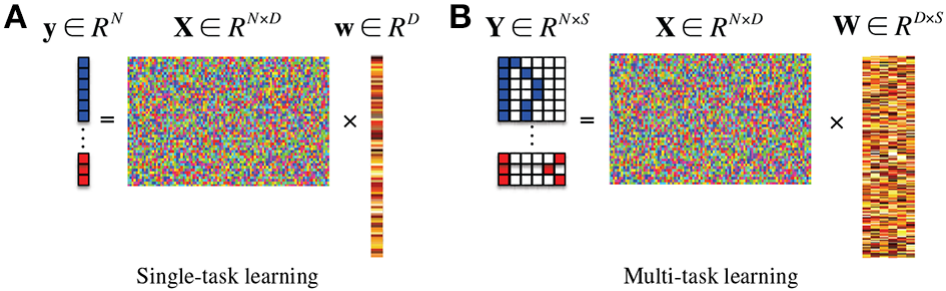
**In the response vector/matrix, the colors of blue, red, and white represent 1, −1, and 0, respectively**. In multi-task learning, each row of the response matrix represents a newly defined sparse code for each sample by the proposed method. **(A)** Single-task learning, **(B)** multi-task learning.

Let **X** ∈ *R*^*N* × *D*^ and **y** ∈ *R*^*N*^ denote, respectively, the *D* neuroimaging features and a clinical label of *N* samples^8^. Assuming that the clinical label can be represented by a linear combination of the neuroimaging features, many research groups have utilized a least square regression model with various regularization terms, which can be mathematically simplified as follows:

(1) $$\underset{w}{\min}\;\|y-Xw{\|}_{F}^{2}+\mathbb{R}(w)$$

where **w** ∈ *R*^*D*^ is a weight coefficient vector and ℝ(**w**) denotes a set of regularization terms. Regarding feature selection, despite its simple form, the ℓ_1_-penalized linear regression model has been widely and successfully used in the literature (Varoquaux et al., 2010; Fazli et al., 2011; de Brecht and Yamagishi, 2012; Suk et al., 2013a), formulated as follows:

(2) $$\underset{w}{\min}\;\|y-Xw{\|}_{F}^{2}+\lambda_{1}\|w{\|}_{1}$$

where λ_1_ denotes a sparsity control parameter. Since the method finds a single optimal weight coefficient vector **w** that regresses the target response vector **y**, it is classified into a single-task learning Figure 1A in machine learning. In this framework, after finding an optimal weight coefficient vector of **w** by means of convex optimization, the features corresponding to zero (or close to zero) weight coefficients are discarded and the remaining ones are considered for the following steps.

If there exists additional class-related information, then we can further extend the ℓ_1_-penalized linear regression model into a more generalized ℓ_2,1_-penalized one Figure 1B (Nie et al., 2010; Cai et al., 2011; Wang et al., 2011) as follows:

(3) $$\underset{W}{\min}\;\|Y-XW{\|}_{F}^{2}+\lambda_{2}\|W{\|}_{2,1}$$

where **Y** ∈ *R*^*N*×*S*^ is a target response matrix, **W** ∈ *R*^*D*×*S*^ is a weight coefficient matrix, *S* is the number of response variables, and λ_2_ denotes a group sparsity control parameter. In machine learning, this framework is classified into a multi-task learning since it needs to find a set of weight coefficient vectors {**w**_1_, …, **w**_*S*_} by regressing multiple response values of **y**_1_, …, **y**_*S*_, simultaneously^9^.

### 3.3. Subclass-based multi-task learning

**Figure 2:**
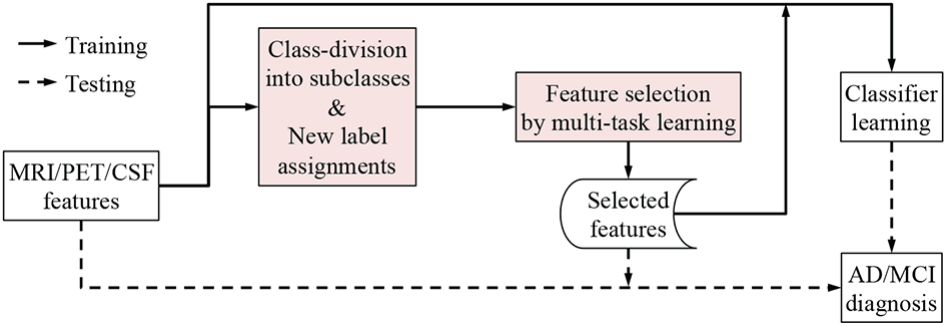
**A framework for AD/MCI diagnosis with the proposed subclass-based multi-task learning**.

We illustrate the proposed framework in Figure 2. In our framework, we first concatenate the multi-modal features into a long vector and then divide each class into a number of subclasses by means of clustering. Based on the clustering results, we encode new class-labels for subclasses and assign them to our training samples. Utilizing the new encoding, a multi-task learning is performed for feature selection. Finally, we train a linear Support Vector Machine (SVM) for classification.

As stated in section 1, it is likely for neuroimaging data to have multiple peaks in distribution due to the inter-subject variability (Fotenos et al., 2005; Noppeney et al., 2006; DiFrancesco et al., 2008). In this paper, we argue that it is necessary to consider the underlying multipeak data distribution in feature selection. To this end, we propose to divide classes into subclasses and to utilize the resulting subclass information in feature selection by means of a multi-task learning.

To divide the training samples in each class to subclasses, we use a clustering technique. Specifically, thanks to its simplicity and computational efficiency, especially in a high dimensional space, we apply a *K*-mean algorithm (Duda et al., 2001). Let *C* = {*c*_*k*_}^*K*^_*k* = 1_ denote a set of *K* clusters and {μ_*k*_}^*K*^_*k* = 1_ be the centers of the clusters (represented by row vectors). Given a set of training samples, the goal of *K*-means algorithm is to minimize the sum of the squared error over all *K* clusters:

(4) $$J(C)=\sum_{k=1}^{K}\sum_{x^{i}\in c_{k}}\|x^{i}-\mu_{k}{\|}^{2}.$$

The main steps of *K*-means algorithm can be summarized as follows (Jain and Dubes, 1988):

1. Initialize a set of *K* cluster means **μ**^(0)^_1_, …, **μ**^(0)^_*K*_.
2. Assignment step: for each of the training samples {**x**^*i*^}^*N*^_*i* = 1_, find a cluster γ^(*t*)^_*i*_ whose mean yields the least Euclidean distance to the sample as follows:
  (5) $$\gamma_{i}^{\left(t\right)}=\underset{c_{k}}{\min}\;\|x^{i}-\mu_{k}^{\left(t-1\right)}{\|}^{2}$$
  where *t* denotes an index of iteration.
3. Update step: for every clusters {*c*_*k*_}^*K*^_*k* = 1_, compute the new mean with the samples assigned to the cluster as follows:
  (6) $$\mu_{k}^{\left(t\right)}=\frac{1}{\left|c_{k}\right|}\sum_{i,\gamma_{i}^{\left(t\right)}=c_{k}}x^{i}$$
  where |*c*_*k*_| denotes the number of samples assigned to the cluster *c*_*k*_ at the iteration *t*.
4. Repeat (2) and (3) until convergence.

After clustering the samples in each class independently, we divide the original classes into their respective subclasses by regarding each cluster as a subclass. We then encode the subclasses with their unique labels, for which we use “discriminative” sparse codes to enhance classification performance. Let *K*_(+)_ and *K*_(−)_ denote, respectively, the number of clusters/subclasses for the original classes of “+” and “−.” Without loss of generality, we define sparse codes for the subclasses of the original classes of “+” and “−” as follows:

(7) $$s_{l}^{\left(+\right)}=\left[\begin{array}{c} +1\;\;\;\;z_{l}^{\left(+\right)}\;\;\;\;0_{K_{\left(-\right)}} \end{array}\right]$$

(8) $$s_{m}^{\left(-\right)}=\left[\begin{array}{c} -1\;\;\;\;0_{K_{\left(+\right)}}\;\;\;\;z_{m}^{\left(-\right)} \end{array}\right]$$

where *l* ∈ {1, …, *K*_(+)_}, *m* ∈ {1, …, *K*_(−)_}, **0**_*K*_(+)__ and **0**_*K*_(−)__ denote, respectively, zero row vectors with *K*_(+)_ and *K*_(−)_ elements, and **z**^(+)^_*l*_ ∈ {0, 1}^*K*_(+)_^ and **z**^(−)^_*m*_ ∈ {0, −1}^*K*_(−)_^ denote, respectively, indicator row vectors in which only the *l*/*m*-th element is set to 1/−1 and the others are 0. Thus, the full code set becomes:

(9) $$S=\{s_{1}^{\left(+\right)},\cdots ,s_{l}^{\left(+\right)},\cdots ,s_{K_{\left(+\right)}}^{\left(+\right)},s_{1}^{\left(-\right)},\cdots ,s_{m}^{\left(-\right)},\cdots ,s_{K_{\left(-\right)}}^{\left(-\right)}\}.$$

For example, assume that we have three and two clusters for “+” and “−” classes, respectively. Then the code set is defined as follows:

(10) $$S=\left\{\begin{array}{cccccccc} s_{1}^{\left(+\right)} & = & [+1 & +1 & 0 & 0 & 0 & 0],\\ s_{2}^{\left(+\right)} & = & [+1 & 0 & +1 & 0 & 0 & 0],\\ s_{3}^{\left(+\right)} & = & [+1 & 0 & 0 & +1 & 0 & 0],\\ s_{1}^{\left(-\right)} & = & [-1 & 0 & 0 & 0 & -1 & 0],\\ s_{2}^{\left(-\right)} & = & [-1 & 0 & 0 & 0 & 0 & -1] \end{array}\right\}.$$

It is noteworthy that in our sparse code set, we reflect the original label information to our new codes by setting the first element of the sparse codes with their original label. Furthermore, by setting the indicator vectors {**z**^(−)^_*m*_}^*K*_(−)_^_*m* = 1_ to be negative, the distances become close among the subclasses of the same original class and distant among the subclasses of the different original classes. That is, in the code set of Equation (10), the squared Euclidean distance between subclasses of the same original class is 2, but that between subclasses of different original classes is 6.

Using the newly defined sparse codes, we assign a new label vector **y**^*i*^ to a training sample **x**^*i*^ as follows:

(11) $$y^{i}=s_{\gamma_{i}}^{\left(y_{i}\right)}$$

where *y*_*i*_ ∈ {+, −} is the original label of the sample **x**^*i*^, and γ_*i*_ denotes the cluster to which the sample **x**^*i*^ was assigned in the *K*-means algorithm. In this way, we extend the original scalar labels of +1 or −1 into sparse code vectors in 𝕊.

Thanks to our new sparse codes, it becomes natural to convert a single-task learning in Equation (2) into a multi-task learning in Equation (3) by replacing the original label vector **y** in Equation (2) with a matrix **Y** = [**y**^*i*^]^*N*^_*i* = 1_ ∈ {−1, 0, 1}^*N* × (1+*K*_(+)_+*K*_(−)_)^ where *K*_(+)_ and *K*_(−)_ denote the number of clusters in the original classes of “+” and “−,” respectively. Figure 1B illustrates the conceptual meaning of our subclass-based multi-task learning, in which the regression of each column vector of **y** is considered as a task. Therefore, we have now (1 + *K*_(+)_ + *K*_(−)_) tasks. Note that the task of regressing the first column response vector **y**_1_ corresponds to our binary classification problem between the original classes of “+” and “−.” Meanwhile, the tasks of regressing the remaining column vectors {**y**_*i*_}^1+*K*_(+)_+*K*_(−)_^_*i* = 2_ formulate new binary classification problems between one subclass and all the other subclasses. It should be noted that unlike the single-task learning that finds a single mapping **w** between regressors **X** and the response **y**, the subclass-based multi-task learning finds multiple mappings {**w**_1_, …, **w**_(1+*K*_(+)_+*K*_(−)_)_}, and thus allows us to efficiently use the underlying multipeak data distribution in feature selection.

### 3.4. Feature selection and classifier learning

Because of the ℓ_2,1_-norm regularizer in our objective function of Equation (3), after finding the optimal solution, we have some zero row-vectors in **W**. In terms of the linear regression, the corresponding features are not informative in regressing the response values. In this regard, we finally select the features whose weight coefficient vector is non-zero, i.e., ‖**w**^*i*^‖_2_ > 0. With the selected features, we then train a linear SVM, which have been successfully used in many applications (Zhang and Shen, 2012; Suk and Lee, 2013).

## 4. Experimental results

### 4.1. Experimental setting

We considered four binary classification problems: AD vs. NC, MCI vs. NC, AD vs. MCI, and MCI-C vs. MCI-NC. In the classifications of MCI vs. NC and AD vs. MCI, we labeled both MCI-C and MCI-NC as MCI. Due to the limited number of samples, we applied a 10-fold cross-validation technique in each binary classification problem. Specifically, we randomly partitioned the samples of each class into 10 subsets with approximately equal size without replacement. We then used 9 out of 10 subsets for training and the remaining one for testing. We reported the performances by averaging the results of 10 cross-validations.

For model selection, i.e., number of clusters *K* in Equation (4), sparsity control parameters of λ_1_ in Equation (2) and λ_2_ in Equation (3), and the soft margin parameter *C* in SVM, we further split the training samples into 5 subsets for nested cross-validation. To be more specific, we defined the spaces of the model parameters as follows: *K* ∈ {1, 2, 3, 4, 5}, *C* ∈ {2^−10^, …, 2^5^}, λ_1_ ∈ {0.001, 0.005, 0.01, 0.05, 0.1, 0.15, 0.2, 0.3, 0.5}, and λ_2_ ∈ {0.001, 0.005, 0.01, 0.05, 0.1, 0.15, 0.2, 0.3, 0.5}. The parameters that achieved the best classification accuracy in the inner cross-validation were finally used in testing. In our implementation, we used a SLEP toolbox^10^ for feature selection and a LIBSVM toolbox^11^ for SVM classifier learning.

To validate the effectiveness of the proposed Subclass-based Multi-Task Learning (SMTL) method, we compared it to the Single-Task Learning (STL) method that used only the original class label as the target response vector in Equation (2). For each set of experiments, we used 93 MRI features, 93 PET features, and/or 3 CSF features as regressors in the respective least square regression models. Regarding the multimodal neuroimaging fusion, e.g., MRI+PET (MP) and MRI+PET+CSF (MPC), we constructed a long feature vector by concatenating features of the modalities. It should be noted that the only difference between the proposed SMTL method and the competing STL method lies in the way of selecting features.

### 4.2. Data distributions

**Figure 3:**
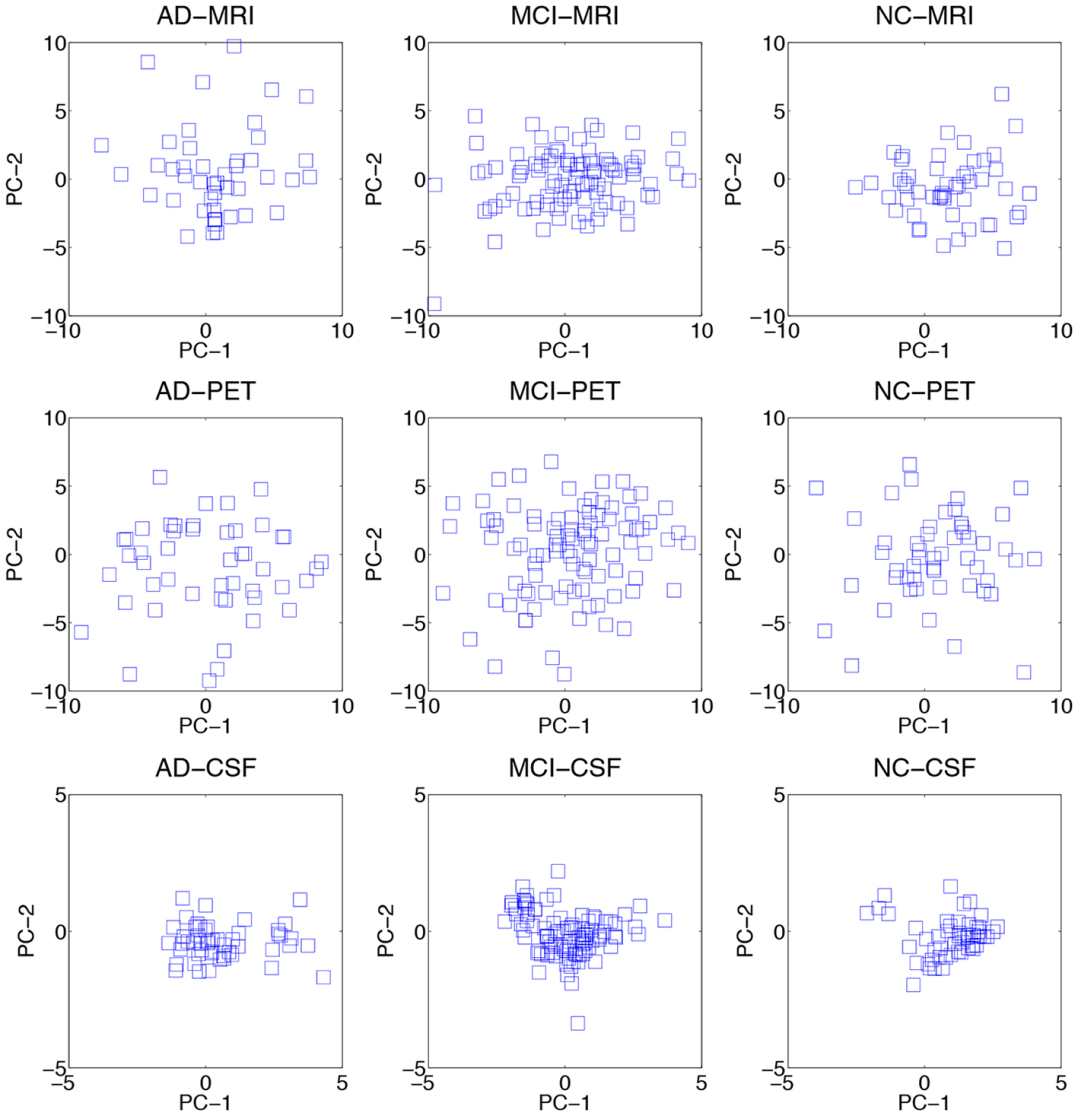
**Data distributions of three modalities over different disease labels**. For visualization, we transformed the original features in an ambient space into their respective 2D eigenspace, whose bases (PC-1 and PC-2) were obtained via principal component analysis.

We visualized the data distributions of our dataset in Figure 3. Due to the high dimensionality of the original feature vectors, we first transformed them into their respective 2D eigenspace, whose bases were obtained via principal component analysis (Duda et al., 2001). From the scatter plots, we can see that most of the data distributions look more like having multiple peaks rather than a single peak. For a quantitative evaluation, we also performed Henze-Zirkler's multivariate normality test (Henze and Zirkler, 1990) and summarized the results in Table 2. In our test, the null hypothesis was that the samples could come from a multivariate normal distribution. Regarding MRI, the null hypothesis was rejected for both AD and MCI. With respect to PET, the test rejected the hypothesis for MCI. In the meantime, it turned out that the CSF samples of all the disease labels didn't follow a multivariate Gaussian distribution. Based on these qualitative and quantitative evaluations, we could confirm the multipeak data distributions and justify the necessity of the subclass-based approach, which can sufficiently handle such multipeak distribution problem.

**Table 2:** **A summary of Henze-Zirkler's multivariate normality test on our dataset**.

**Modality**	**AD**	**MCI**	**NC**
MRI	0.0005 (R)	0.0004 (R)	0.6967 (A)
PET	0.4273 (A)	0.0239 (R)	0.3150 (A)
CSF	0.0049 (R)	<0.0001 (R)	<0.0001 (R)

“R” or “A” in parentheses denotes whether the null hypothesis (that the samples could come from a multivariate normal distribution) is rejected or accepted at the 5% significance level.

### 4.3. Performance measurements

Let TP, TN, FP, and FN denote, respectively, True Positive, True Negative, False Positive, and False Negative. In our experiments, we considered the following five metrics:

- ACCuracy (ACC) = (TP+TN) / (TP+TN+FP+FN).
- SENsitivity (SEN) = TP / (TP+FN).
- SPECificity (SPEC) = TN / (TN+FP).
- Balanced ACcuracy (BAC) = (SEN+SPEC) / 2.
- Area Under the receiver operating characteristic Curve (AUC).

The accuracy that counts the number of correctly classified samples in a test set is the most direct metric for comparison between methods. Regarding the sensitivity and specificity, the higher the values of these metrics, the lower the chance of mis-diagnosing. Note that in our dataset, in terms of the number of samples available for each class, they are highly imbalanced, i.e., AD(51), MCI(99), and NC(52). Therefore, it is likely to have an inflated performance estimates for the classifications of MCI vs. NC and AD vs. MCI. For this reason, we also consider a balanced accuracy that considers the imbalance of a test set. Lastly, one of the most effective measurements of evaluating the performance of diagnostic tests in brain disease as well as other medical areas is the Area Under the receiver operating characteristic Curve^12^ (AUC). The AUC can be thought as a measure of the overall performance of a diagnostic test. The larger the AUC, the better the overall performance of the diagnostic test.

### 4.4. Classification results

We summarized the performances of the competing methods with various modalities for AD and NC classification in Table 3. The proposed method showed the mean ACCs of 93.27% (MRI), 89.27% (PET), 95.18% (MP), and 95.27% (MPC). Compared to the STL method that showed the ACCs of 90.45% (MRI), 86.27% (PET), 92.27% (MP), and 94.27% (MPC), the proposed method improved by 2.82% (MRI), 3% (PET), 2.91% (MP), and 1% (MPC) in accuracy. The proposed SMTL method achieved higher AUC values than the STL method for all the cases. It is also remarkable that, except for the metric of specificity with PET, 90.33% (STL) vs. 88.33% (SMTL), the proposed method consistently outperformed the competing STL method over all the metrics and modalities.

**Table 3:** **A summary of the performances for AD vs. NC classification**.

**Method**	**Modality**	**ACC (%)**	**SEN (%)**	**SPEC (%)**	**BAC (%)**	**AUC (%)**
STL
	MRI	90.45±6.08	82.67	**98.33**	90.50	93.55
	PET	86.27±8.59	82.00	90.33	86.17	90.12
	MP	92.27±5.93	90.00	94.67	92.33	94.91
	MPC	94.27±6.54	**94.00**	94.33	94.17	95.74
SMTL
	MRI	93.27±6.33	88.33	**98.33**	93.33	94.19
	PET	89.27±7.43	90.00	88.33	89.17	91.67
	MP	95.18±6.65	**94.00**	96.33	**95.17**	96.15
	MPC	**95.27**±**6.58**	**94.00**	96.33	**95.17**	**97.13**

(STL, Single-Task Learning; SMTL, Subclass-based Multi-Task Learning). The boldface denotes the best performance in each metric.

In the discrimination of MCI from NC, as reported in Table 4, the proposed method showed the ACCs of 76.82% (MRI), 74.18% (PET), 79.52% (MP), and 80.07% (MPC). Meanwhile, the STL method showed the ACCs of 74.85% (MRI), 69.51% (PET), 74.85% (MP), and 76.82% (MPC). Again, the proposed method outperformed the STL method by improving ACCs of 1.97% (MRI), 4.67% (PET), 4.67% (MP), and 3.25% (MPC), respectively. It is believed that the high sensitivities and the low specificities for both competing methods resulted from the imbalanced data between MCI and NC. In the metrics of BAC and AUC that somehow reflect the imbalance of the test samples, the proposed method achieved the best BAC of 77.06% and the best AUC of 81.82% with MPC.

**Table 4:** **A summary of the performances for MCI vs. NC classification**.

**Method**	**Modality**	**ACC (%)**	**SEN (%)**	**SPEC (%)**	**BAC (%)**	**AUC (%)**
STL
	MRI	74.85±5.92	80.67	64.00	72.33	76.55
	PET	69.51±10.11	74.78	59.67	67.22	73.54
	MP	74.85±3.91	84.78	56.00	70.39	78.79
	MPC	76.82±7.15	85.89	59.33	72.61	79.25
SMTL
	MRI	76.82±7.15	85.78	59.67	72.72	77.84
	PET	74.18±7.18	81.89	59.67	70.78	72.73
	MP	79.52±5.39	**88.89**	62.00	75.44	77.91
	MPC	**80.07**±**8.42**	86.78	**67.33**	**77.06**	**81.82**

(STL, Single-Task Learning; SMTL, Subclass-based Multi-Task Learning). The boldface denotes the best performance in each metric.

From a clinical point of view, establishing the boundaries between preclinical AD and mild AD, i.e., MCI, has practical and economical implications. To this end, we also performed experiments on AD vs. MCI classification and summarized the results in Table 5. Similar to the MCI vs. NC classification, because of the imbalanced data, we had a large gap between sensitivities and specificities. Nevertheless, the proposed method still showed the best ACC of 74.60%, the best BAC of 67.83%, and the best AUC of 72.85% with MP.

**Table 5:** **A summary of the performances for AD vs. MCI classification**.

**Method**	**Modality**	**ACC (%)**	**SEN (%)**	**SPEC (%)**	**BAC (%)**	**AUC (%)**
STL
	MRI	62.68±7.01	4.00	93.00	48.50	59.16
	PET	72.02±6.73	31.33	93.00	62.17	69.50
	MP	69.26±8.66	51.00	78.56	64.78	71.40
	MPC	68.40±14.48	41.33	82.44	61.89	70.19
SMTL
	MRI	70.60±5.97	39.00	86.67	62.83	66.90
	PET	73.31±3.25	33.00	**94.00**	63.50	67.78
	MP	**74.60**±**9.57**	**46.67**	89.00	**67.83**	**72.85**
	MPC	72.60±9.88	37.33	**91.00**	64.17	71.74

(STL, Single-Task Learning, SMTL, Subclass-based Multi-Task Learning). The boldface denotes the best performance in each metric.

Lastly, we conducted experiments of MCI-C and MCI-NC classification, and compared the results in Table 6. The proposed SMTL method achieved the best ACC of 72.02%, the best BAC of 70.33%, and the best AUC of 69.64% with MP. In line with the fact that the classification between MCI-C and MCI-NC is the most important for early diagnosis and treatment, it is remarkable that compared to the STL method, the ACC improvements by the proposed method were 4.62% (MRI), 5.15% (PET), 7.4% (MP), and 7.22% (MPC), respectively.

**Table 6:** **A summary of the performances for MCI-C vs. MCI-NC classification**.

**Method**	**Modality**	**ACC (%)**	**SEN (%)**	**SPEC (%)**	**BAC (%)**	**AUC (%)**
STL
	MRI	56.98±20.61	51.00	60.67	55.83	58.85
	PET	61.58±17.79	55.00	66.00	60.50	60.63
	MP	64.62±14.04	62.50	66.00	64.25	63.87
	MPC	62.89±12.29	58.50	66.00	62.25	58.31
SMTL
	MRI	61.60±13.12	44.00	75.67	59.83	60.76
	PET	66.73±11.32	39.00	**88.00**	63.50	65.57
	MP	**72.02**±**13.80**	58.00	82.67	**70.33**	**69.64**
	MPC	70.11±14.21	**59.00**	78.67	68.83	67.36

(STL, Single-Task Learning, SMTL, Subclass-based Multi-Task Learning). The boldface denotes the best performance in each metric.

In order to further verify the superiority of the proposed SMTL method compared to the STL method, we also performed a statistical significance test to assess whether the differences in classification ACCs between the methods are at a significant level on the dataset by means of a paired *t*-test. Here, the null hypothesis in our work was that the proposed SMTL method produced the same mean ACCs as the STL method. The *p*-values were 8.884e-04 (AD vs. NC), 4.85e-05 (MCI vs. NC), 1.11e-03 (AD vs. MCI), 7.48e-03 (MCI-C vs. MCI-NC), respectively. That is, the proposed SMTL method statistically outperformed the STL method for all the cases, rejecting the null hypothesis beyond the 95% confidence level.

### 4.5. Discussion

**Figure 4:**
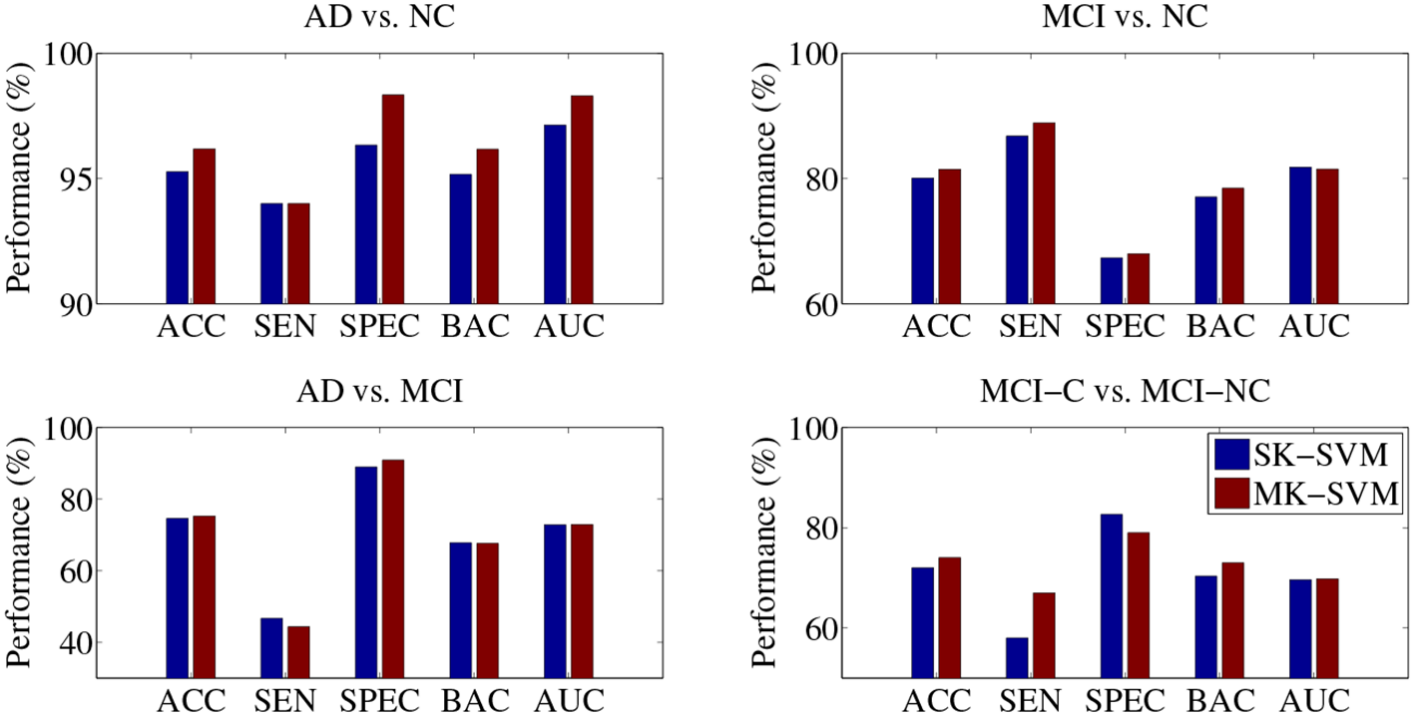
**Performance comparison between SK-SVM and MK-SVM in four binary classifications**. For both methods, the feature selection was performed on the concatenated feature vectors with the proposed subclass-based multi-task learning.

In the classifications of AD vs. MCI and MCI-C vs. MCI-NC, the proposed SMTL method with MP, rather than with MCP, achieved the best performances. That is, although we used richer information with MPC, i.e., additional CSF features, the performances with MPC were lower than with MP in those classification problems. Based on the results, fusing the CSF features with the other modalities turned out to be a confounding factor in the classifications of AD vs. MCI and MCI-C vs. MCI-NC. Furthermore, in our experiments above, the selected features were fed into a SVM classifier and in this stage, the features of different modalities have equal weights in decision, which can be a potential problem degrading the performances. To this end, we additionally performed experiments by replacing a Single-Kernel linear SVM (SK-SVM) with a Multi-Kernel linear SVM (MK-SVM) (Gönen and Alpaydin, 2011), with which we could find optimal weights for the modalities. The modality weights were determined by nested cross-validation similarly for model parameters selection described in section 4.1. Specifically, we applied a grid search with an interval of 0.1 with the constraint of the sum of the modality weights to be one. In Figure 4, we compared the best performances of SK-SVM, i.e., equal weights for modalities, with those of MK-SVM. It should be noted that for both methods of SK-SVM and MK-SVM, we applied the proposed STML method for feature selection. By means of a modality-adaptive weighting strategy with MK-SVM, we obtained the maximal ACCs of 96.18% (AD vs. NC), 81.45% (MCI vs. NC), 73.21% (AD vs. MCI), and 74.04% (MCI-C vs. MCI-NC). That is, MK-SVM clearly outperformed the SK-SVM by improving the ACCs of 0.91% (AD vs. NC), 1.41% (MCI vs. NC), 0.67% (AD vs. MCI), and 2.02% (MCI-C vs. MCI-NC), respectively.

In Table 7, we also compared the classification accuracies of the proposed method with those of the state-of-the-art methods that fused multimodal neuroimaing for the classifications of AD vs. NC and MCI vs. NC. Note that, due to different datasets and different approaches of extracting features and building classifiers, it may not be fair to directly compare the performances among the methods. Nevertheless, the proposed method showed the highest accuracies among the methods in both classification problems. In particular, it is noteworthy that compared to Zhang and Shen's work (Zhang et al., 2011) in which they used the same dataset with ours, the proposed method enhanced the accuracies by 2.98 and 5.05% for the classifications of AD vs. NC and MCI vs. NC, respectively. Furthermore, in comparison with Liu et al.'s work (Liu et al., 2013), where they used the same types of features from MRI and PET and the same number of subjects with ours, our method improved the accuracies by 1.81% (AD vs. NC) and 2.65% (MCI vs. NC), respectively.

**Table 7:** **Comparison of classification accuracies with the state-of-the-art methods that used multimodal neuroimaing for AD/MCI vs. NC**.

**Methods**	**Subjects (AD**/**MCI**/**NC)**	**Modality**	**AD vs. NC (%)**	**MCI vs. NC (%)**
Kohannim et al., 2010	40/83/43	MRI+PET+CSF	90.7	75.8
Hinrichs et al., 2011	48/119/66	MRI+PET	92.4	n/a
Zhang et al., 2011	51/99/52	MRI+PET+CSF	93.2	76.4
Westman et al., 2012	96/162/111	MRI+CSF	91.8	77.6
Liu et al., 2013	51/99/52	MRI+PET	94.37	78.80
Proposed method	51/99/52	MRI+PET+CSF	**96.18**	**81.45**

The boldface denotes the best performance in each classification task.

**Figure 5:**
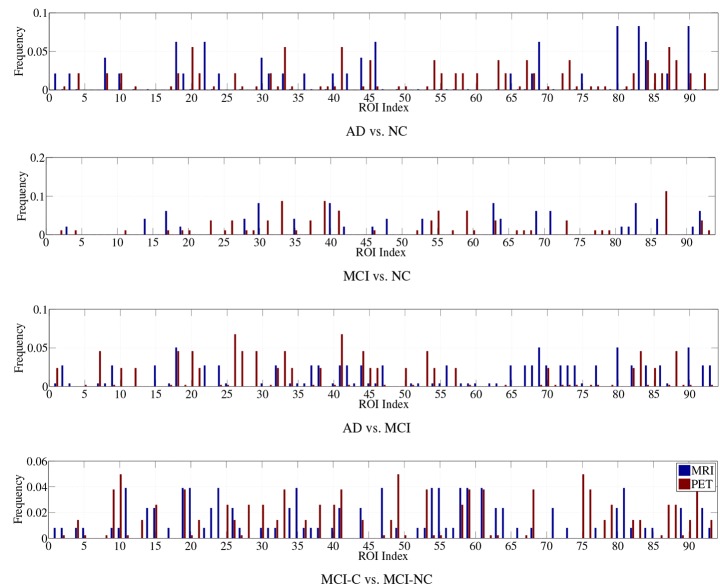
**Normalized histograms of the selected features in four binary classification problems**.

**Figure 6:**
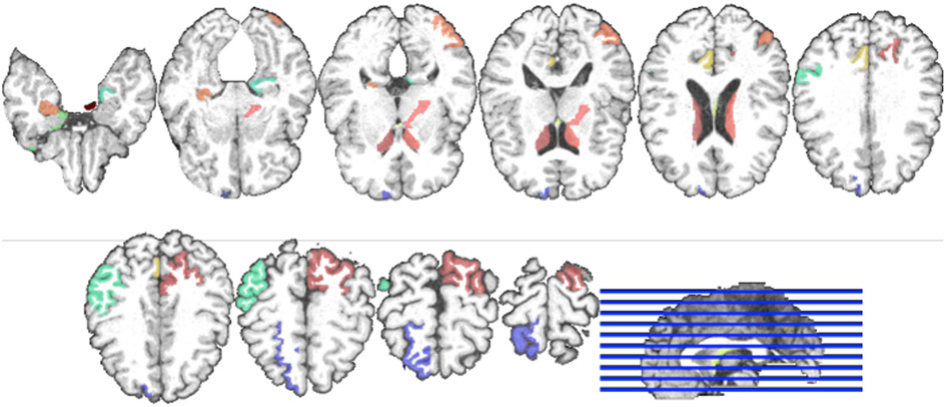
**Visualization of the selected ROIs by the proposed method**. Different colors denote different brain areas.

Regarding the interpretation of the selected ROIs, due to the involvement of cross-validation, multimodal neuroimaging fusion, and multiple binary classifications in our experiments, it was not straightforward to analyze the selected ROIs. In this work, we first built a histogram of the frequency of the selected ROIs of MRI and PET over cross-validations per binary classification, and normalized it by considering only the ROIs whose frequency was larger than the mean frequency and set the frequency of the disregarded ROIs to zero. Figure 5 presents the normalized frequency of the selected ROIs in each binary classification. We then added the four normalized histograms in Figure 5 to find the relative frequency of the selected ROIs over four classification problems. We finally selected ROIs whose frequency was larger than the mean normalized frequency and visualized them in Figure 6. Those ROIs include amygdala, hippocampus, parahippocampal gyrus (Braak and Braak, 1991; Visser et al., 2002; Mosconi, 2005; Lee et al., 2006; Devanand et al., 2007; Burton et al., 2009; Desikan et al., 2009; Walhovd et al., 2010; Ewers et al., 2012), superior frontal gyrus, insula, anterior/posterior cingulate gyrus, inferior occipital gyrus, post central gyrus, supramarginal gyrus (Buckner et al., 2005; Desikan et al., 2009; Dickerson et al., 2009; Schroeter et al., 2009), precuneus, paracentral lobule (Bokde et al., 2006; Singh et al., 2006; Davatzikos et al., 2011), heschl gyrus (Supekar et al., 2008), superior/middle temporal gyrus, temporal pole, inferior temporal (Chan et al., 2001; Visser et al., 2002; Burton et al., 2009).

## 5. Conclusions

In this paper, we proposed a novel method that formulates a subclass-based multi-task learning. Specifically, to take into account the underlying multipeak data distribution of the original classes, we applied a clustering method to partition each class into multiple clusters, which further considered as subclasses. Here, we can think that one cluster, i.e., subclass, represents one peak in distribution. The respective subclasses were encoded with their unique codes, for which we imposed the subclasses of the same original class close to each other and those of different original classes distinct from each other. We assigned the newly defined codes to our training samples as new label vectors and applied a ℓ_2,1_-norm regularizer in a linear regression framework, thus formulated a multi-task learning problem. We finally selected features based on the optimal weight coefficients. It is noteworthy that unlike the previous methods of PCA, LDA, and other embed methods for dimensionality reduction, the proposed method considered multiple mapping functions to reflect the underlying multipeak data distributions, and thus to enhance performances in AD/MCI diagnosis. In our experimental results on the publicly available ADNI dataset, we proved the validity of the proposed method by outperforming the competing methods in four binary classifications of AD vs. NC, MCI vs. NC, AD vs. NC, and MCI-C vs. MCI-NC.

In the context of the practical application of the proposed method, it should be considered for how to determine the optimal number of clusters, i.e., *K*, for each class, although, in this paper, we applied a cross-validation technique for dealing with this issue. One potential solution for this issue is to use affinity propagation algorithm (Frey and Dueck, 2007) that does not require the number of clusters to be determined. The other potential limitation of our work is that outliers or contaminated features could affect our clustering results, thus causing performance degradation by selecting uninformative features or unselecting informative features. All these limitations will be considered in our future research.

### Conflict of interest statement

The Reviewer Dr. Heng Huang declares that, despite having collaborated with the authors, the review process was handled objectively and no conflict of interest exists. The authors declare that the research was conducted in the absence of any commercial or financial relationships that could be construed as a potential conflict of interest.

## Footnotes

^2^Available online at “http://adni.loni.usc.edu/”

^3^Although there exist in total more than 800 subjects in ADNI database, only 202 subjects have the baseline data including all the modalities of MRI, FDG-PET, and CSF.

^4^Refer to “http://www.adni-info.org/Home.aspx” for more details.

^5^Available online at “http://mipav.cit.nih.gov/clickwrap.php”

^6^Available online at “http://fsl.fmrib.ox.ac.uk/fsl/fslwiki/”

^7^While the most intuitive feature should be the voxel in MRI and FDG-PET, due to their extremely high dimensionality, in this paper, we take a ROI-based approach and consider the GM tissue volumes and the mean intensity for each ROI from MRI and FDG-PET, respectively, as the features. Furthermore, by using the ROI-based features for our classification, the performances can be less affected by the partial volume effect in PET imaging (Aston et al., 2002).

^8^In this work, we have one sample per subject and consider a binary classification.

^9^To regress each response value is considered as a task.

^10^Available online at “http://www.public.asu.edu/~jye02/Software/SLEP/index.htm.”

^11^Available online at “http://www.csie.ntu.edu.tw/~cjlin/libsvm/.”

^12^The receiver operating characteristic curve is defined as a plot of test true positive rate vs. its false positive rate.
